# Early Recovery of Sympathetic Ophthalmia with Hearing Loss in a Young Man

**DOI:** 10.7759/cureus.1814

**Published:** 2017-11-02

**Authors:** Tan Chew-Ean, Khairuddin Othman, Sheena Mary Alexander, Ismail Shatriah

**Affiliations:** 1 Department of Ophthalmology, School of Medical Sciences, University Science Malaysia, Kubang Kerian, Kelantan, Malaysia.; 2 Department of Ophthalmology, Queen Elizabeth Hospital, Kota Kinabalu, Sabah, Malaysia.

**Keywords:** sympathetic ophthalmia, hearing loss, early recovery

## Abstract

Hearing loss is rarely associated with sympathetic ophthalmia. We describe a young man who presented with sympathetic ophthalmia and concurrent hearing loss one month post globe rupture. The presentation was very subtle and atypical. However, the patient recovered fully after two weeks of prompt oral corticosteroid therapy which resulted in good visual and hearing outcomes. This patient demonstrated that the acute phase of sympathetic ophthalmia is reversible with early recognition of features, timely diagnosis, and rapid initiation of corticosteroid therapy.

## Introduction

Sympathetic ophthalmia refers to a diffuse granulomatous uveitis in both eyes. The occurrence of sympathetic ophthalmia had been reported in up to 3.1% of patients with a history of trauma to one eye [1]. It is proclaimed to develop within five days to 66 years [2]. About 70% to 80% of the cases occur within three months of ocular insult, while 90% of cases occur within one year. Sympathetic ophthalmia can have atypical associating systemic presentations such as vitiligo, poliosis, alopecia, dysacusis, and meningeal irritation [3].

Hearing loss is a rare clinical manifestation of sympathetic ophthalmia. It is more likely to occur in Vogt-Koyanagi-Harada syndrome. We describe a case of sympathetic ophthalmia with concurrent hearing loss which presented at the early stage and was treated to full recovery within two weeks.

## Case presentation

A 35-year-old Kadazan man had a firecracker injury and sustained rupture of the left globe with full thickness scleral laceration from the 12 to 6 o’clock position, uveal prolapse, and total hyphema. A skull x-ray was performed, which revealed no bony fracture or radio-opaque foreign body. He underwent a primary scleral repair in our institution. Postoperatively, the visual acuity of the left eye was no perception to light. He was prescribed guttate moxifloxacin 0.5% every two hours, guttate prednisolone every four hours, and guttate homatropine 2%  every eight hours in the injured eye. Intravenous ciprofloxacin 400 mg 12 hourly was completed for one week.

After two weeks, the injured eye became phthisical, and the cornea became opaque. The visual acuity of the right eye was 6/7.5. The anterior segment examination was normal with unremarkable fundus findings. One month later, the patient presented with a history of sudden onset of central scotoma in the right eye for three days. It was associated with difficulty in reading and tinnitus in the left ear. The patient denied symptoms of eye redness, pain, or photophobia. There was no preceding fever, neck pain, headache, or vertigo noted.

**Figure 1:**
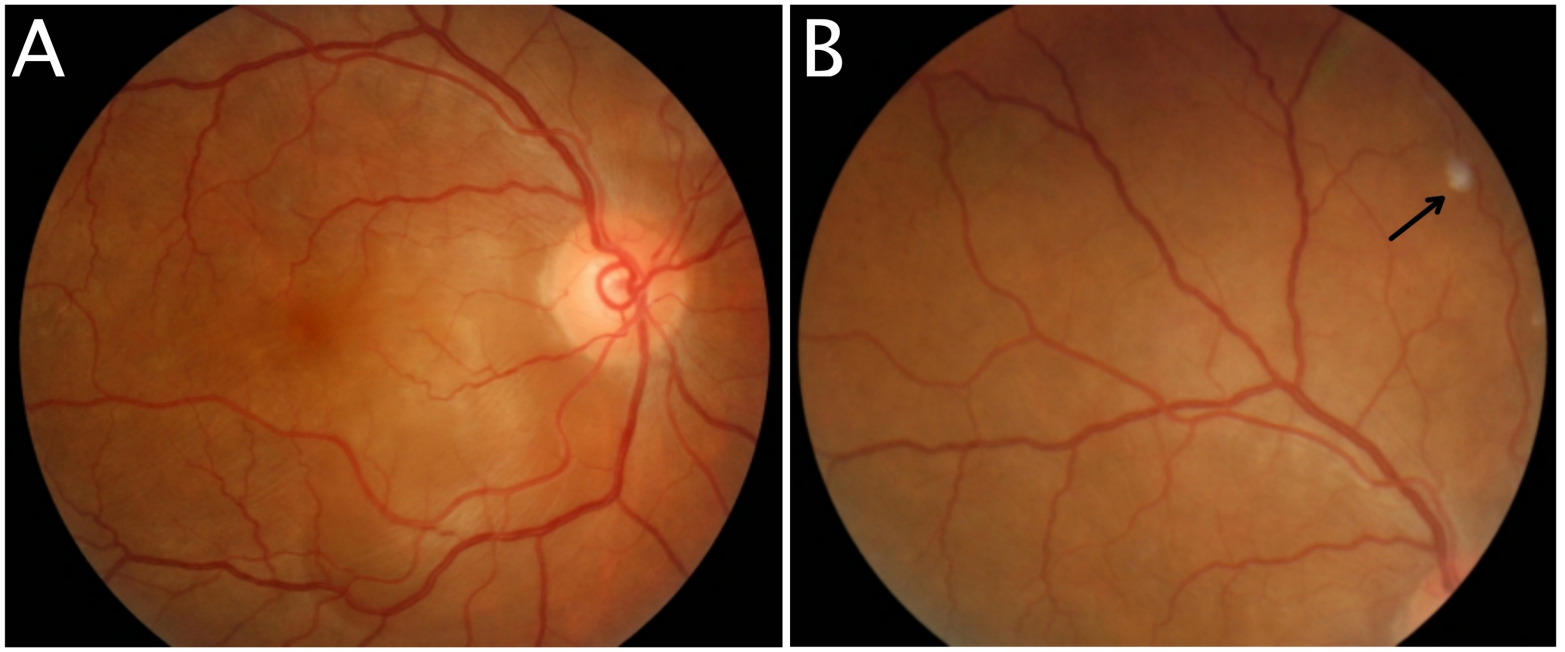
Fundus photography of right eye Focal serous retinal detachment located at the nasal and inferior areas of the macula during presentation (1A). Dalen Fuch nodule (arrow) at superonasal quadrant of the retina (1B).

Visual acuity of the right eye was 6/7.5. The anterior segment examination showed the presence of cells 2+ with granulomatous keratic precipitates. The fundus examination revealed a focal serous retinal detachment located at the nasal and inferior areas of the macula. No vitritis was noted. The optic disc was pink and non-swollen (Figure 1A). A single Dalen Fuch nodule was observed at the superonasal area of the retina (Figure 1B).

**Figure 2:**
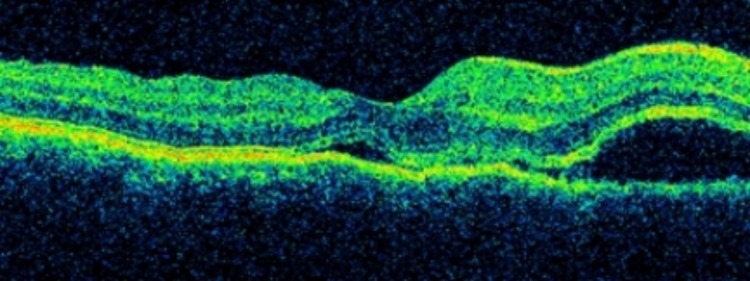
Optical coherence tomography of right macula Subretinal fluid accumulated at the macula during presentation.

The visual acuity of the left eye remained no perception of light. The left cornea was decompensated and hazy. Hence, it obscured a proper assessment of the anterior and posterior segments of the left eye. The intraocular pressure was 14 mmHg in the right eye and 9 mmHg in the left eye. Optical coherence tomography of right macula showed the presence of subretinal fluid in the macula (Figure 2). Fundus fluorescein angiography was not performed in this patient.

**Figure 3:**
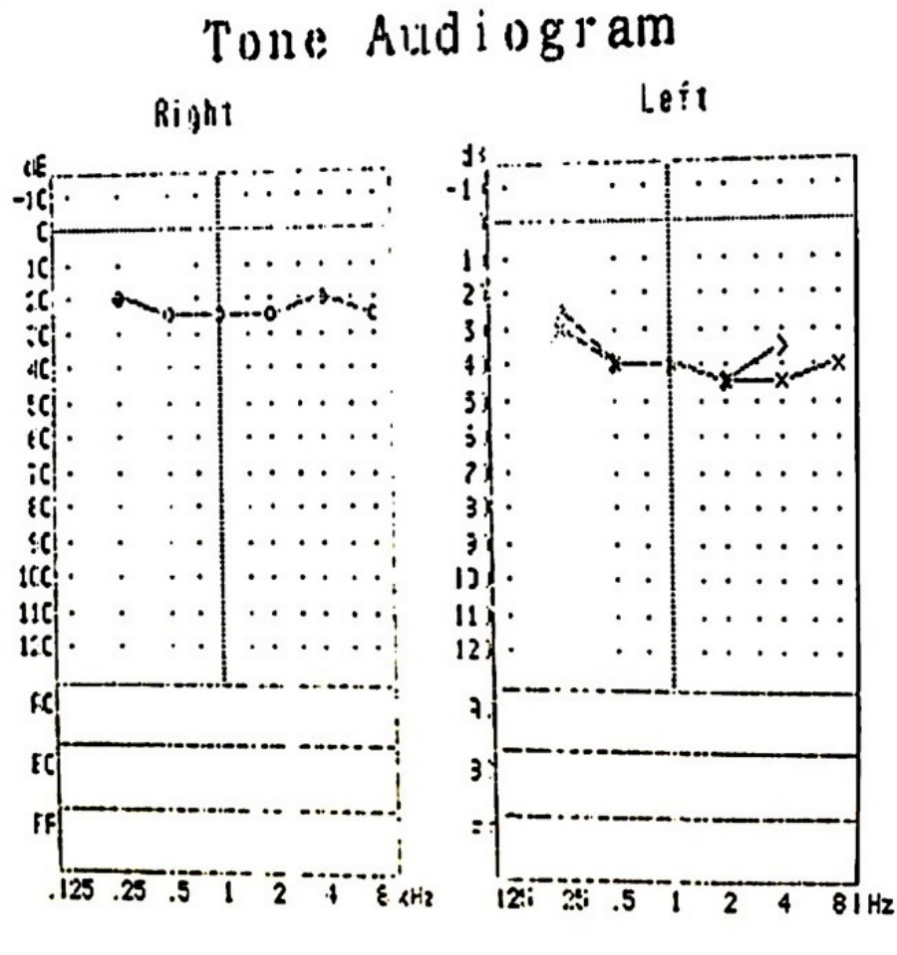
Pure tone audiogram Pure tone audiogram before treatment showing left sensorineural hearing loss.

Systemic and neurological examination findings were normal. He was referred to an otolaryngology specialist for a hearing assessment. Tympanogram was bilaterally normal. Pure tone audiometry showed sensorineural deafness in the left ear (Figure 3). Hearing assessment of the right ear was normal. Blood and urine cultures did not reveal any growth, the erythrocyte sedimentation rate (ESR) was 1 mm/hour, Mantoux test was 3x3 mm induration, and the x-ray of the chest was normal. Other blood investigations for infective causes including venereal disease research laboratory (VDRL) and (human immunodeficiency virus) HIV screenings were reported negative.

**Figure 4:**
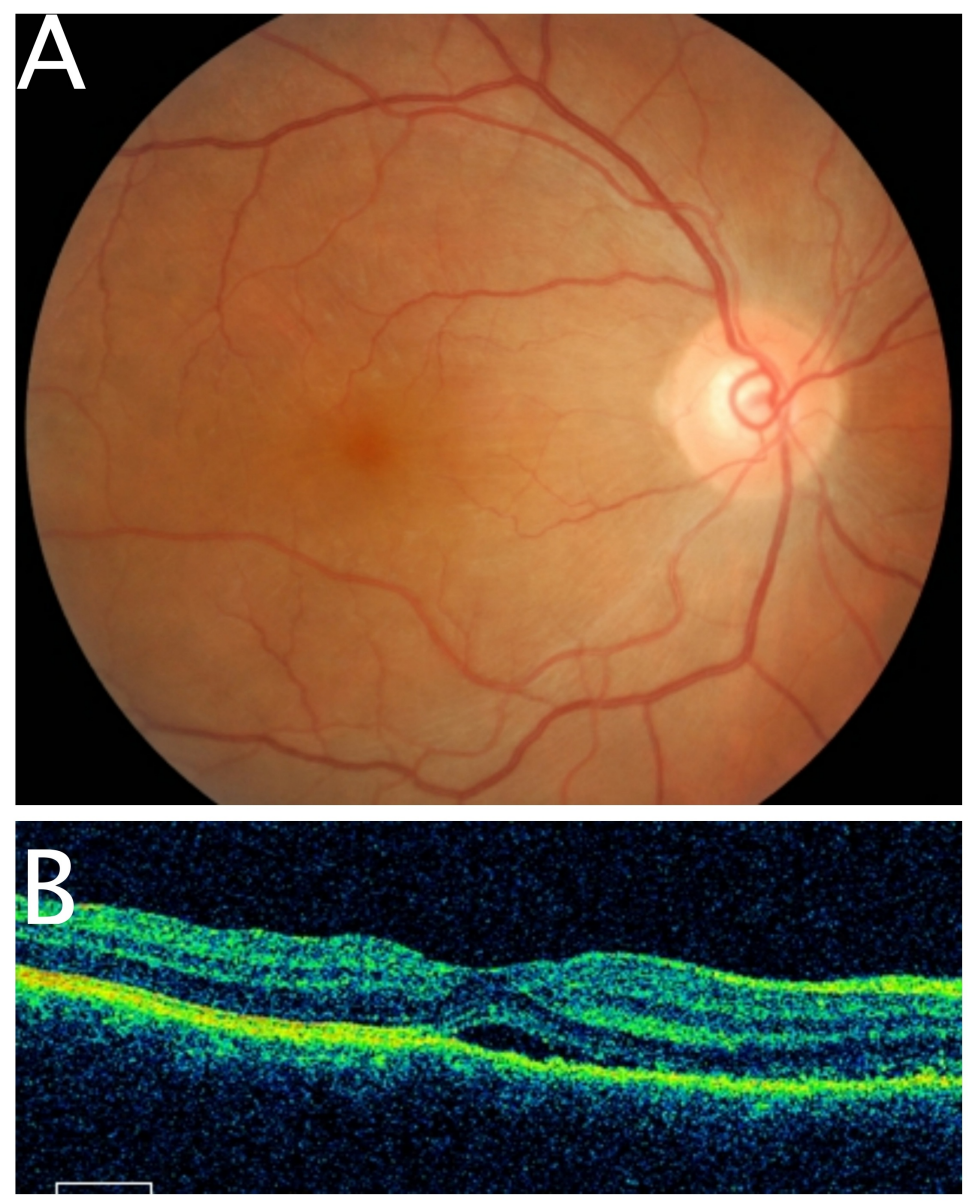
Fundus photography and optical coherence tomography of right eye Tremendous recovery after five days of treatment evidenced by minimal serous retinal detachment (4A). A rapid reduction in subretinal fluid collection (4B).

He was diagnosed with sympathetic ophthalmia and was prescribed guttate dexamethasone 0.1% four hourly, guttate homatropine 2% eight hourly, and oral prednisolone 1 mg/kg daily. Five days after starting oral prednisolone, the visual acuity of the right eye improved from 6/7.5 to 6/6. Anterior chamber cells were 1+ and the fundus examination revealed minimal serous retinal detachment at the macula (Figure 4A). Repeat optical coherence tomography of the right macula demonstrated a rapid reduction of subretinal fluid collection (Figure 4B). Oral prednisolone was tapered weekly after clinical resolution of sympathetic ophthalmia was observed.

**Figure 5:**
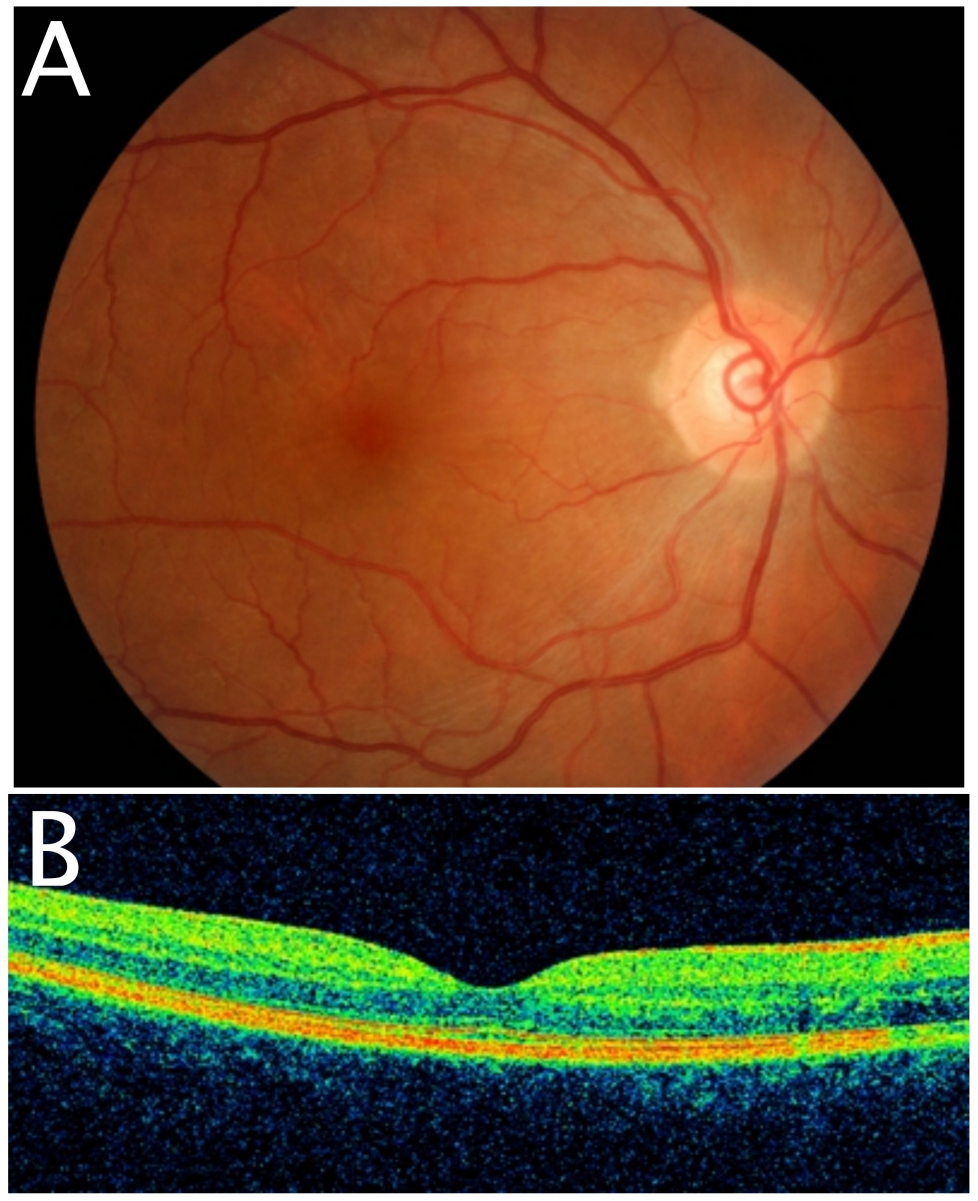
Fundus photography and optical coherence tomography of right eye Complete clinical resolution of serous retinal detachment after two weeks of treatment (5A). Total resorption of subretinal fluid was observed with optical coherence tomography (5B).

Follow-up at the second week demonstrated complete recovery from sympathetic ophthalmia in the right eye. The visual acuity stabilized at 6/6, and the anterior segment was quiet. Fundus assessment showed a normal looking macula with resolved Dalen Fuch nodule (Figure 5A). Optical coherence tomography of the right macula confirmed a complete regression of subretinal fluid collection (Figure 5B).

**Figure 6:**
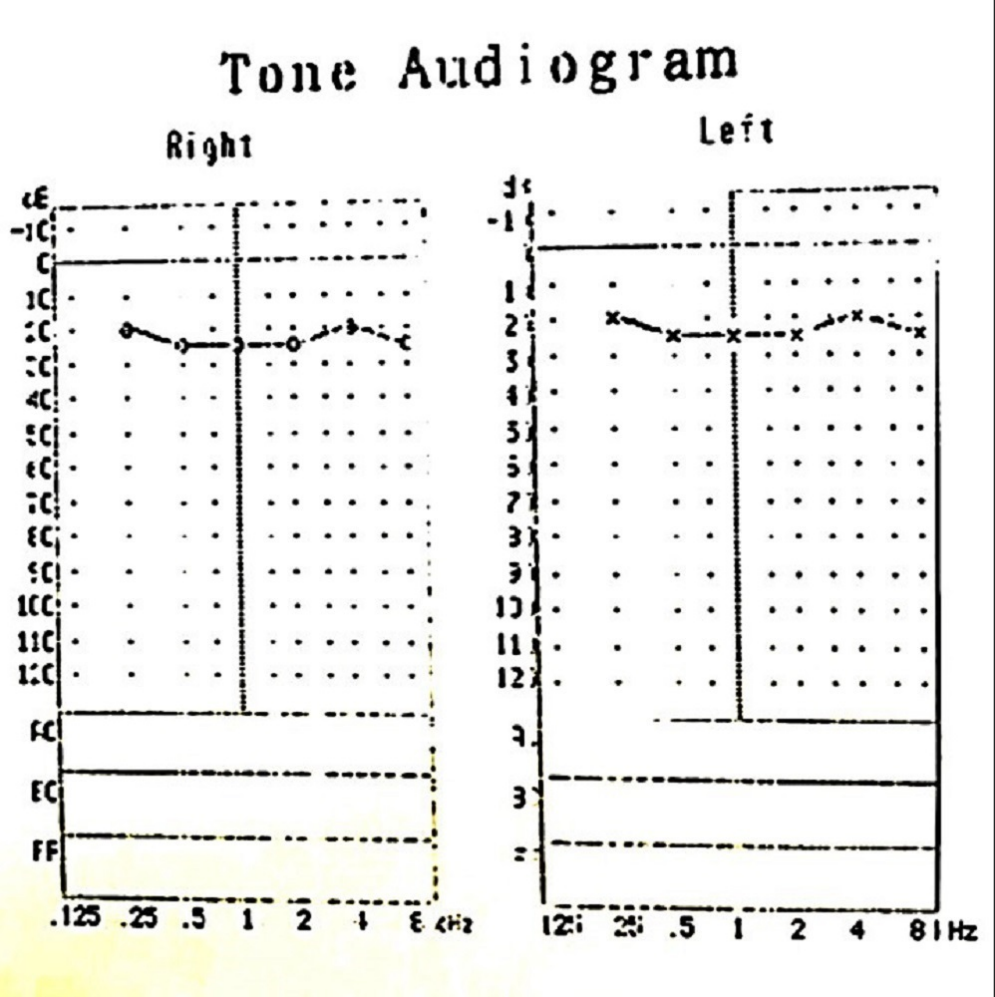
Pure tone audiogram Repeat pure tone audiogram at two months after treatment showing full recovery of hearing loss in the left ear.

He was reviewed at two months and six months follow-up. Visual acuity in the right eye remained 6/6. Ocular examination showed no signs suggestive of recurrence. Hearing loss in the left ear recovered two months after treatment. Repeat pure tone audiometry revealed no recurrence (Figure 6). Oral prednisolone was stopped two months later after a gradual tapering regime.

## Discussion

Hearing loss is an unusual presentation in sympathetic ophthalmia. It is believed to involve a T-cell-mediated autoimmune response to melanocytes. These melanocytes are found in the uveal tract and the intermediate cell layer of stria vascularis of the cochlear duct. Sensorineural hearing loss can be a transient or permanent sequelae [4].

There were few reported cases describing sympathetic ophthalmia and concurrent hearing loss in the literature [4-7]. Table 1 summarizes these published cases of sympathetic ophthalmia and hearing loss, including our patient. Most patients presented at 4–12 weeks after an ocular insult. Four out of five patients had ocular trauma, while one occurred following a vitreo-retina surgery. Their presentation was poor visual acuity, panuveitis, dense vitritis, and bilateral hearing loss.

**Table 1:** Published literature on sympathetic ophthalmia with hearing loss and including our patient RE: right eye, LE: left eye, BE: both eyes, IOFB: intraocular foreign body, VR: vitreoretina, MH: macula hole, RD: retinal detachment, SO: sympathetic ophthalmia, BOV: blurring of vision, HL: hearing loss, VA: visual acuity, NPL: non-perception to light, PL: perception of light, HM: hand movement, CF: counting finger, NA: not available, ORL: otorhinolaryngology, SNHL: sensorineural hearing loss, IVMP: intravenous methyprednisolone, PO: per os, by mouth or orally

Authors	Year	Age/ Gender	Risk Factors	Onset SO After Insult	Presenting Symptoms	Eye Findings		ORL Findings	Treatment	Duration of Recovery	Final Outcome	
						RE	LE				Visual Recovery	Hearing Recovery
Nirankari, et al. [5]	1970	55 / Male	LE penetrating injury	2 months	RE BOV & bilateral HL 20 days	VA: PL, panuveitis	VA: NPL, phthisis bulbi, corneoscleral tear, uveal prolapse	Bilateral HL	RE retrobulbar injection dexamethasone then PO dexamethasone	2 months	Yes. RE 6/36	No
Comer, et al. [6]	2005	72 / Female	LE trabeculectomy then LE globe rupture	25 days	RE BOV & bilateral HL	VA: 6/24, panuveitis (dense vitritis)	VA: HM, total hyphema, uveal prolapse	Bilateral HL	IVMP 1 g then PO prednisolone 20 mg daily, cyclosporine, LE pars plana lensectomy & vitrectomy	3 months	Yes. RE 6/6, LE 6/12	No
Venkatesh, et al. [7]	2013	23 / Male	RE penetrating injury with IOFB	3 months	BE BOV & bilateral HL	VA: CF, anterior uveitis, hyperemic disc	VA: PL, panuveitis (dense vitritis), exudative RD	Bilateral SNHL	IVMP 1.5 mg/kg 3 days then PO corticosteroid 1.5 mg/kg, azathioprine, topical prednisolone	15 months	Yes. RE 6/36, LE 6/9	Yes
Kawashima, et al. [4]	2015	80 / Female	RE penetrating injury	25 days	Bilateral HL 5 days & BE BOV	VA: NA, panuveitis	VA: NA, panuveitis	Bilateral moderate SNHL	IVMP 1 g 3 days then PO prednisolone 1 mg/kg for 6 months	1 month	Yes	Yes
		32 / Female	RE VR surgery 2 times for myopic MH & RD	43 days	BE BOV & bilateral HL	VA: NA, panuveitis	VA: NA, panuveitis	Bilateral mild SNHL	IVMP 1 g 3 days then PO prednisolone 1 mg/kg for 6 months	1 month	Yes	Yes
Our patient	2017	35 / Male	LE globe rupture	1 month	RE BOV 3 days & Left HL 1 week	VA: 6/7.5, anterior uveitis, subretinal fluid	VA: NPL, phthisis bulbi	Left SNHL	PO prednisolone 1mg/kg with tapering dose for 2 months, topical dexamethasone	2 weeks	Yes. RE 6/6	Yes

Our patient had a similar onset of presentation at four weeks after an incident of left globe rupture. He presented with features of granulomatous anterior uveitis, good visual acuity, and unilateral sensorineural hearing loss. Our patient's presentation is more subtle compared to the other patients who reported the same symptoms [4-7]. This is probably because our patient presented at the early stage of the attack. The circulating T-cells mediated autoimmune attack melanocytes in both ears without preference of laterality, which probably explains the occurrence of contralateral hearing loss in our patient. 

Systemic corticosteroid is the mainstay treatment of sympathetic ophthalmia. Immunosuppressive agents such as cyclosporine and azathioprine provide good control of inflammation while avoiding the undesirable systemic side-effects of prolonged usage of corticosteroid. Intravitreal triamcinolone acetonide is another option for treatment, but frequent injections can result in cataract formation and steroid-induced glaucoma [8]. Newer treatment modalities, such as an intravitreal fluocinolone acetonide implant, have demonstrated excellent control of non-infectious uveitis [8-9]. 

All of the reported patients had more severe presentations and were started with intravenous methylprednisolone. In contrast, our patient was only prescribed oral prednisolone. Three patients with hearing loss recovered well after corticosteroid therapy; two of them improved after one month and another patient recovered at 15 months [4,7]. The remaining two patients had permanent hearing loss [5-6]. This may suggest that patients who had mild to moderate hearing loss recovered well with appropriate treatment. Our patient achieved complete resolution of vision and hearing impairment at the second week of treatment, which was earlier than expected. The earliest ever reported recovery is at one month post-treatment.

Close monitoring is critical in the 4-12 weeks after ocular trauma or surgery.This is essential for early detection of subtle presentation of sympathetic ophthalmia. Thus, an appropriate treatment can be administered early with the aim of improving the prognosis and final outcome. Our patient's presentation is not a classical manifestation of sympathetic ophthalmia. However, early diagnosis and adequate corticosteroid treatment demonstrated a successful recovery of his vision and hearing. 

## Conclusions

Diagnosis of early and atypical presentation of sympathetic ophthalmia is challenging. High index of suspicion is crucial for early identification and accurate diagnosis. Early initiation of corticosteroid therapy can result in complete resolution of the acute phase of sympathetic ophthalmia with a good final outcome.
